# Subcellular localization of nucleocapsid protein of SFTSV and its assembly into the ribonucleoprotein complex with L protein and viral RNA

**DOI:** 10.1038/s41598-021-01985-x

**Published:** 2021-11-26

**Authors:** Sithumini M. W. Lokupathirage, Yoshimi Tsuda, Kodai Ikegame, Kisho Noda, Devinda S. Muthusinghe, Fumiya Kozawa, Rashid Manzoor, Kenta Shimizu, Kumiko Yoshimatsu

**Affiliations:** 1 Graduate School of Infectious Diseases, Hokkaido University, Sapporo, 060-8638 Japan; 2 Department of Microbiology and Immunology, Faculty of Medicine, Hokkaido University, Sapporo, 060-8638 Japan; 3 Graduate School of Medicine, Hokkaido University, Sapporo, 060-8638 Japan; 4 School of Medicine, Hokkaido University, Sapporo, 060-8638 Japan; 5 International Institute for Zoonosis Control, Hokkaido University, Sapporo, 001-0020 Japan; 6 Institute for Genetic Medicine, Hokkaido University, Sapporo, 060-0815 Japan

**Keywords:** Virus-host interactions, Cellular microbiology

## Abstract

Severe fever with thrombocytopenia syndrome virus (SFTSV) is an emerging bunyavirus that causes novel zoonotic diseases in Asian countries including China, Japan, South Korea, and Vietnam. In phleboviruses, viral proteins play a critical role in viral particle formation inside the host cells. Viral glycoproteins (GPs) and RNA-dependent RNA polymerase (RdRp) are colocalized in the Golgi apparatus and endoplasmic reticulum-Golgi intermediate compartment (ERGIC). The nucleocapsid (N) protein was widely expressed in the cytoplasm, even in cells coexpressing GP. However, the role of SFTSV N protein remains unclear. The subcellular localization of SFTSV structural proteins was investigated using a confocal microscope. Subsequently, minigenome and immunoprecipitation assays were carried out. The N protein interacts with viral RNA (vRNA) and further shows translational activity with RdRp which is L protein and localized in the ERGIC and Golgi apparatus when co-expressed with GP. On the other hand, mutant N protein did not interact with vRNA either localized in the ERGIC or Golgi apparatus. The interaction between the N protein of SFTSV and vRNA is important for the localization of viral proteins and viral assembly. This study provides useful insights into the life cycle of SFTSV, which will lead to the detection of antiviral targets.

## Introduction

Severe fever with thrombocytopenia syndrome (SFTS) is an emerging zoonosis caused by the SFTS virus (SFTSV), which belongs to the genus *Bandavirus* in the family *Phenuiviridae* order *Bunyavirales* according to the International Committee on Taxonomy of Viruses classification criteria. SFTS was first reported in Hubei Province in China; in Japan, it was first identified in Yamaguchi Prefecture in 2013^1,2^. SFTS patients have been reported in South Korea and Vietnam, and SFTS is now considered as an infectious disease widely distributed in East Asia^3,4^. SFTS is a tick-borne zoonotic disease characterized by fever, gastrointestinal symptoms, thrombocytopenia, and leukopenia^5^. The fatality rate of SFTS in Japan is 6–30%, while the fatality rate in other countries is 12–47%^6–8^. Since effective drugs and vaccines have not yet been developed, it is essential to understand the viral replication mechanism to develop antiviral agents against SFTS.

The RNA genome of SFTSV is tripartite, negative or ambi-sense, single-stranded RNA designated as small, medium (M), and large (L) segments, which encode for the nucleocapsid (N) protein and the non-structural protein, glycoprotein (GP); Gn and Gc, and L protein which has main role on RNA-dependent RNA polymerase (RdRp) activity, respectively. The bunyaviruses enter the cell by binding to receptors such as C-type lectin on dendritic cells via the GP, and then the virus is taken up into the cells by clathrin-dependent endocytosis^9,10^. As a result, the ribonucleoprotein (RNP) complex is released into the cell cytoplasm, which causes transcription and replication of viral genomic RNA (vRNA). N protein and RdRp are expressed in the cytoplasm where they form the RNP complex with the vRNA^11^. Gn and Gc, which are translated in the endoplasmic reticulum (ER), associate with the RNP complex in the ER-Golgi intermediate compartment (ERGIC) or Golgi apparatus, followed by budding at the membranes of the Golgi apparatus^12,13^. Finally, Golgi vesicles containing virus particles are trafficked to the cell surface and release infectious virions extracellularly via exocytosis.

We previously reported that SFTSV structural proteins were localized together in the ERGIC and Golgi apparatus in SFTSV-infected cells. In cells transfected with all viral proteins, GP localized to the ER, ERGIC, and Golgi apparatus, but N protein or RdRp alone did not localize to any cellular compartment. In co-transfection experiments with GP, a small amount of RdRp, but not N protein, changed the localization of GP to the ERGIC and Golgi apparatus^13^. In this study, we aimed to elucidate the mechanism of the localization of N protein to the ERGIC and Golgi apparatus, which is imperative for virus particle formation in the replication cycle of SFTSV.

## Results

### Localization of N protein in the presence of RdRp and GP in transfected cells

**Figure 1:**
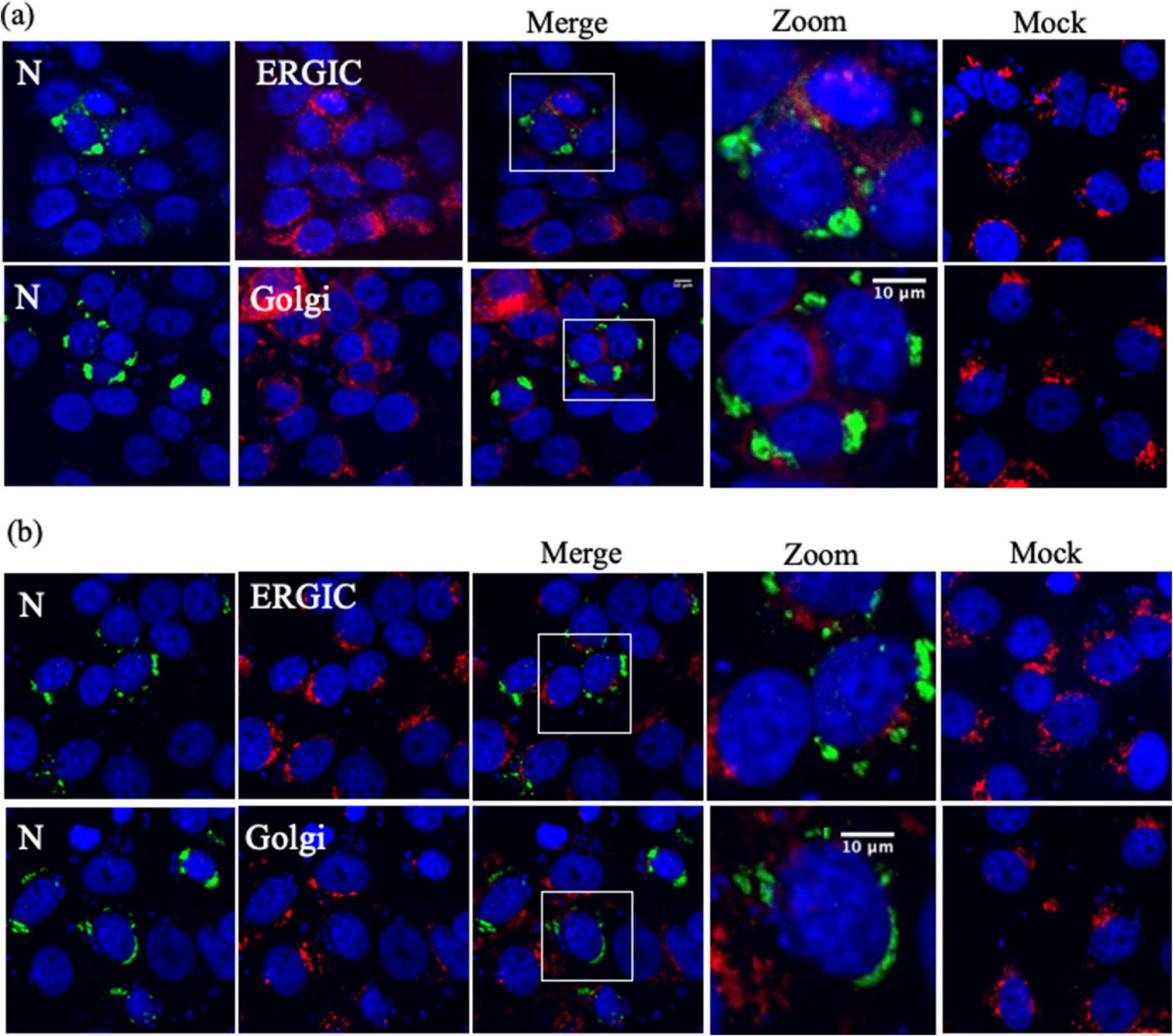
Subcellular localization of N proteins with GP or RdRp. **(a)** Effect of GP and RdRp expression on the localization of N in Vero E6 cells. Vero E6 cells were transfected with pCAGGS-SFTSV-N, pCAGGS-SFTSV-GP, and pCAGGS-SFTSV-RdRp and subsequently fixed at 24 h post-transfection (hpt). **(b)** Effect of RdRp and vRNA-Rluc on the localization of N in Vero E6 cells. Vero E6 cells were transfected with pCAGGS-SFTSV-N, pCAGGS-SFTSV-RdRp, and vRNA-Rluc and subsequently fixed at 24 hpt. The cells were stained with antibodies against N protein (green) and ERGIC or Golgi organelle markers (red), and the nuclei were stained with DAPI (blue). Magnification of the merged area is shown on the right side of the merged image. The mock-transfected cells are shown as mock images.

Interaction with GP is an important factor for the localization of viral components into the ERGIC and Golgi apparatus, which is a viral assembly compartment; Lundu et al. reported the localization of GP in the presence of N protein. In this study, we reproduced the same results using N protein and GP, especially focusing on the N protein (Supplementary Fig. 1). N protein and GP were co-expressed in Vero E6 cells, and the intracellular localization of N protein was analyzed under a confocal microscope. The N protein was not localized to the ERGIC or Golgi apparatus in the presence of GP, which agrees with the results of previous studies. To further clarify the relationship between GP and RdRp, we carried out a subcellular localization experiment in which GP and RdRp were co-expressed. RdRp was localized to the ERGIC and Golgi apparatus in the presence of GP, which agrees with the results of Lundu et al. (Supplementary Fig. 2a)^13^. In Supplementary Fig. 2b, we estimated the colocalization using the Pearson’s correlation coefficients (PCCs), which is used to calculate the number of respective coefficients. The PCC value range differs from − 1.0 to 1.0, where 0 indicates no significant correlation and 1 indicates a complete positive correlation^14^. The number of cells showing high PCC values in RdRp localization in the presence of GP to the ERGIC was higher than that to the Golgi apparatus. Previous studies reported that the GP of SFTSV is expressed in the ER of infected cells and then moves to the Golgi apparatus via the ERGIC and accumulates in the Golgi apparatus, which suggests that GP plays a major role in leading RdRp to the budding compartments^13^. It is thought that both N protein and RdRp are synthesized in the cytoplasm and then germinate together with mature GP in the ERGIC to the Golgi apparatus to form viral particles. Next, Vero E6 cells were transfected simultaneously with plasmids of N protein, GP, and RdRp, and the localization of N protein to the ERGIC and Golgi apparatus was examined (Fig. 1a). Even in cells simultaneously co-expressing RdRp and GP, the N protein did not localize to the budding compartments. These results suggest that viral factors other than viral structural proteins are required for viral particle formation. Therefore, to understand the role of vRNA in viral particle formation and interaction of N protein with vRNA and RdRp, we used cells that co-expressed the N protein, RdRp, and vRNA-Rluc (which mimics vRNA), and analyzed the localization of N protein to the ERGIC and Golgi apparatus (Fig. 1b). In the presence of RdRp and vRNA-Rluc, N protein was detected in the cytoplasm, but translocation to the ERGIC and Golgi apparatus was not detected.

### Localization of N protein to the ERGIC and Golgi apparatus in cells expressing all viral structural components

**Figure 2:**
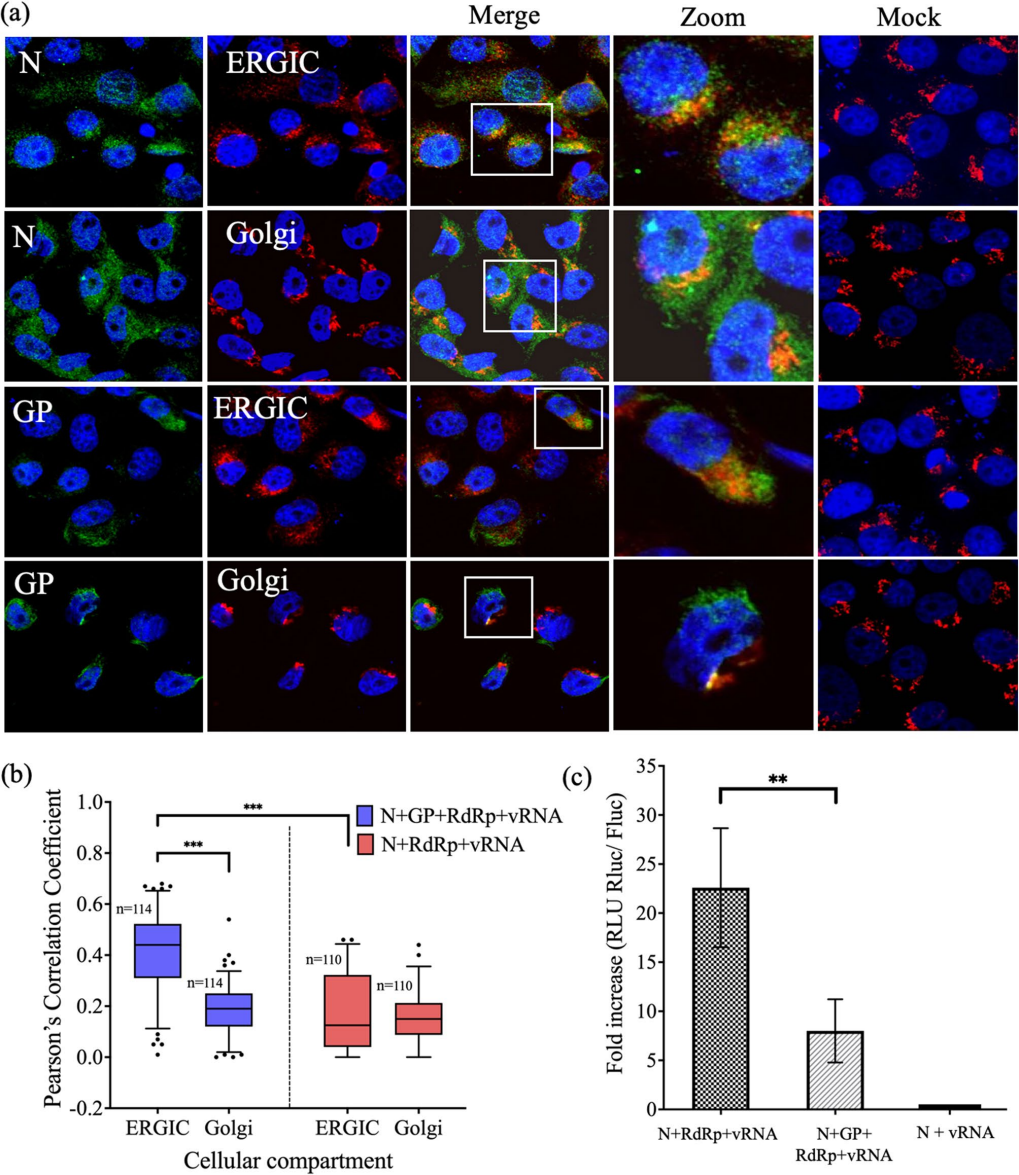
Subcellular localization of N protein together with all viral components. **(a)** Vero E6 cells were transfected with pCAGGS-SFTSV-N, pCAGGS-SFTSV-GP, pCAGGS-SFTSV-RdRp, and vRNA-Rluc and subsequently fixed at 24 hpt. The cells were stained with antibodies against N protein or GP (green) and ERGIC or Golgi organelle markers (red), and the nuclei were stained with DAPI (blue). The yellow areas in the merged images show the cellular localization of proteins with organelle markers. Magnification of the merged area is shown on the right side of the merged image. The mock-transfected cells are shown as mock images. **(b)** Localization of N, ERGIC, and Golgi apparatus shown in **(a)** was analyzed using ImageJ (corresponding to * P < 0.05, ** P < 0.01, and *** P < 0.001). Number of cells = n. **(c)** Detection of transcriptional activity using a minigenome assay. BHK/T7-9 cells were transfected with pCAGGS-SFTSV-N, pCAGGS-SFTSV-RdRp, and vRNA-Rluc or pCAGGS-SFTSV-N, pCAGGS-SFTSV-RdRp, vRNA-Rluc, and pCAGGS-SFTSV-GP. The luciferase activities were compared with that of N + vRNA-Rluc (without RdRp) as a negative control. The activity of Rluc was standardized by Fluc activity (transfection control). Results are shown as fold increases (RLU Rluc/Fluc) in the bar graph with standard deviations of the means. The statistical significance between RdRp and without RdRp in different combinations was assessed using the Student’s t-test (corresponding to *P < 0.05 and ** P < 0.01).

Subcellular localization experiments were carried out using all viral structural components, including N protein, GP, RdRp, and vRNA-Rluc. Cellular localization of N protein and GP to the ERGIC and Golgi apparatus in the presence of all viral components was confirmed (Fig. 2a). Similarly, Lundu et al. reported that N protein, GP, and RdRp fluorescent signals colocalized with those of ERGIC and Golgi markers in SFTSV-infected cells^13^. A significant difference was observed in the PCC value between the presence and absence of GP. Interestingly, the incidence of N protein localization to the ERGIC was higher than that to the Golgi apparatus in the presence of GP (Fig. 2b). These results suggest that N protein, GP, RdRp, and vRNA are essential for viral assembly, as was previously shown for hantavirus virion assembly^15^.

A minigenome assay was performed by transfecting cells with the N protein, RdRp, and vRNA-Rluc. Luciferase minigenome activity was detected (Fig. 2c), indicating that N proteins assemble into RNPs together with RdRp and vRNA, which mimic the RNP complex in the cell cytoplasm. This finding demonstrates that the expressed N protein functionally interacts with RdRp and vRNA in the cell cytoplasm. However, subcellular localization experiments did not confirm the localization of N protein to the ERGIC and Golgi apparatus in cells expressing all viral proteins, except for GPs (Fig. 1b). Interestingly, a minigenome assay with GP showed decreased luciferase activity compared with that in the minigenome assay of N protein and RdRp, which exhibited the highest luciferase activity (Fig. 2c).

### Interaction of the N protein with vRNA is essential for N protein.

**Figure 3:**
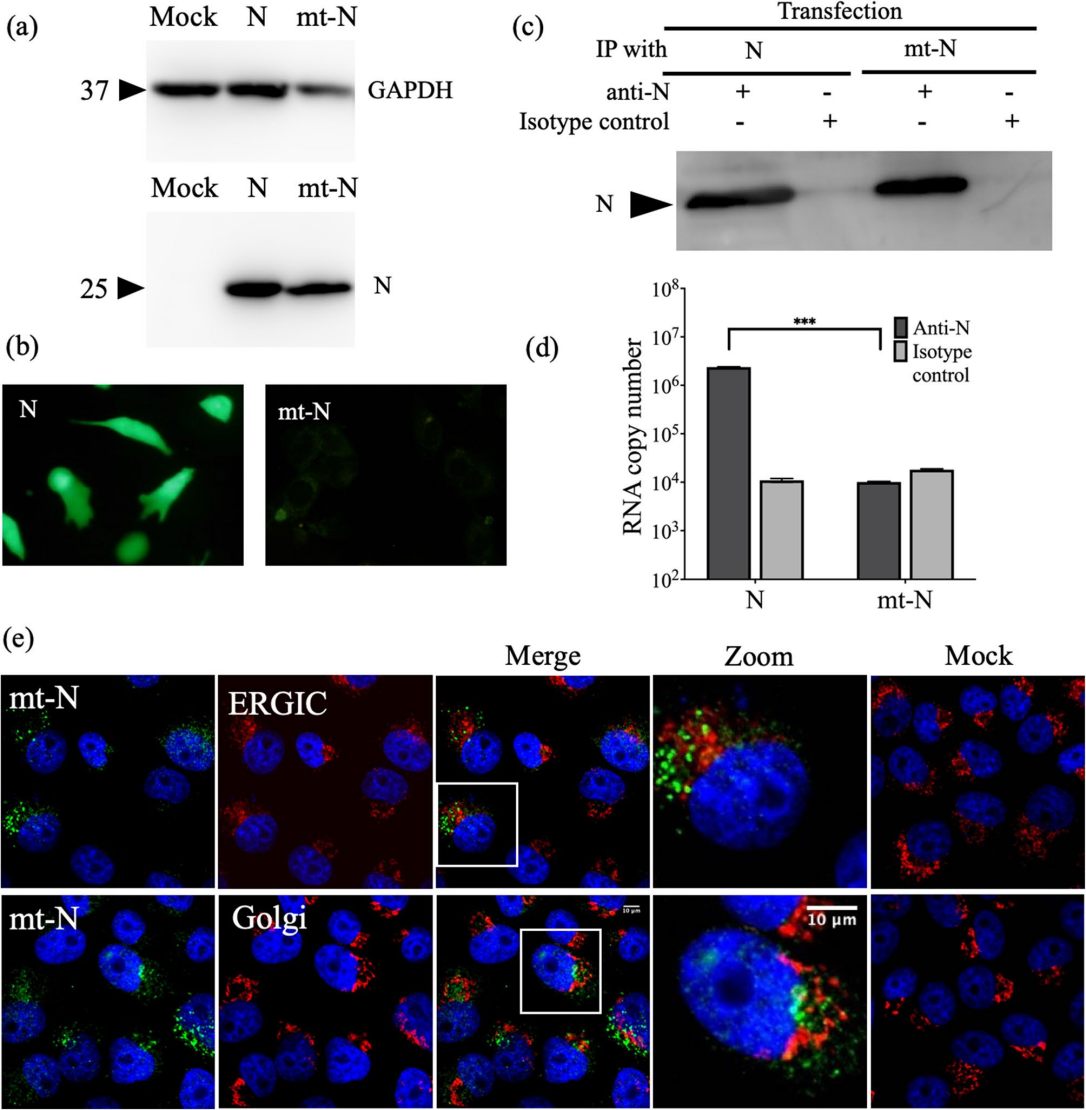

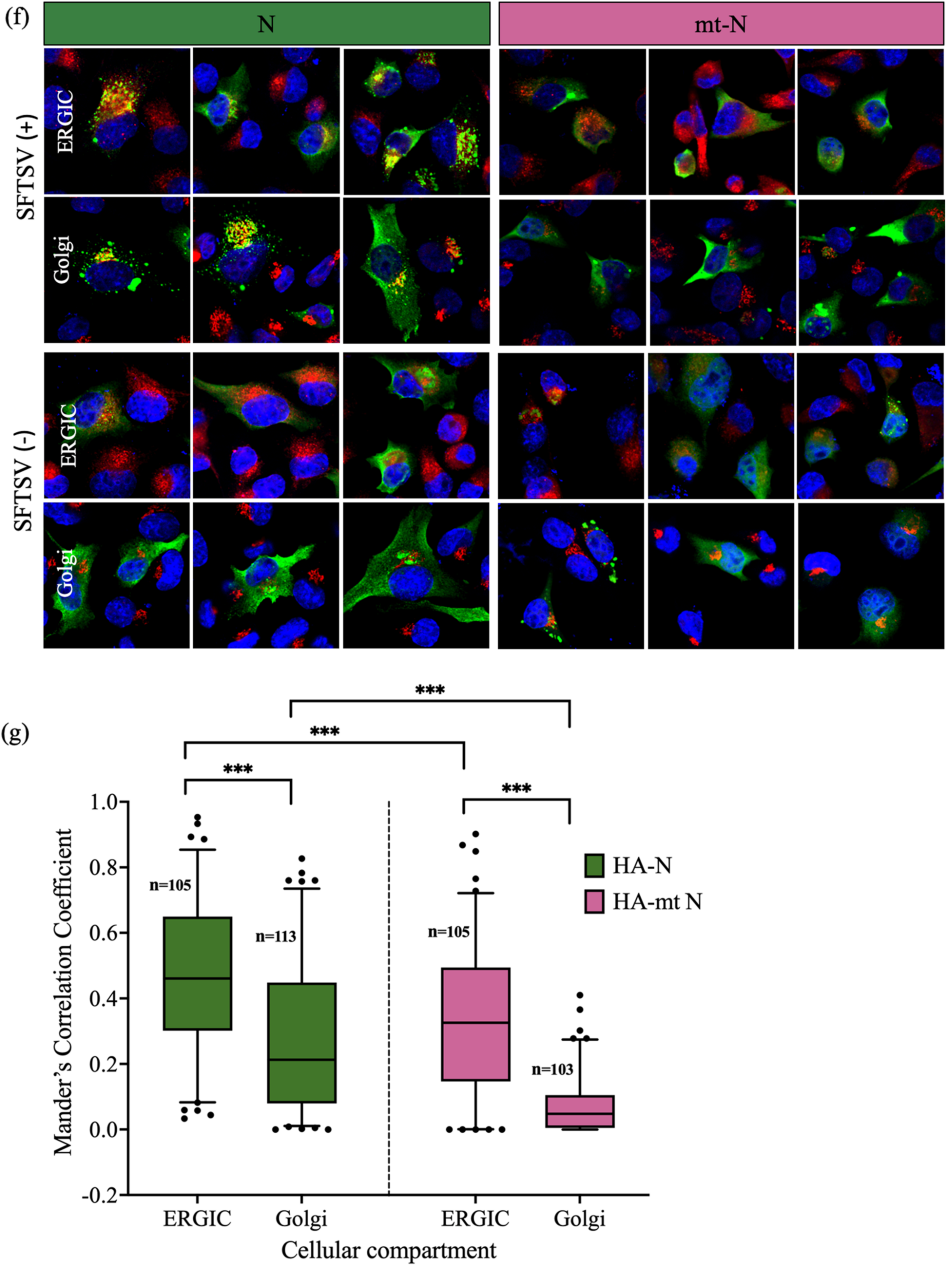
Functional analysis of N and mt-N proteins of SFTSV. **(a)** The cell lysate of mt-N protein-transfected 293 T cells was used for western blot analysis, and expressed proteins were determined using anti-rabbit N polyclonal antibody as the primary antibody and HRP-conjugated Protein A as the secondary antibody. GAPDH was used as a control. **(b)** BSR-T7/5 cells were transfected with pCAGGS-SFTSV N (left panel) or pCAGGS-SFTSV mt-N (Right panel) together with pCAGGS-SFTSV L and pATX-vRNA-eGFP and eGFP expression was examined. **(c)** Cell lysates from **(b)** were immunoprecipitated with anti-N. Both N and mt-N proteins successfully bound to the beads and were detected by western blotting using rabbit anti-N pAb. **(d)** Minigenome RNA was detected from immunoprecipitated fraction by qPCR of eGFP gene (corresponding to * P < 0.05, ** P < 0.01, and *** P < 0.001). **(e)** Subcellular localization of the mt-N protein in Vero cells. Plasmids pCAGGS-SFTSV-mt-N, pCAGGS-SFTSV-RdRp, pCAGGS-SFTSV-GP, and vRNA-Rluc were transfected, and the luciferase activity was not detected in these cells (Supplementary Fig. 5). The cells were stained with antibodies against N protein (green) and ERGIC or Golgi organelle markers (red), and the nuclei were stained with DAPI (blue). Magnification of the merged area is shown on the right side of the merged image. The mock-transfected cells are shown as mock images. **(f)** Sub-cellular localization of HA-tagged N and HA-tagged mt-N proteins in SFTSV-infected BHK/T7-9 cells. SFTSV-infected BHK/T7-9 cells were transfected with pCAGGS-SFTSV-N or pCAGGS-SFTSV mt-N. SFTSV infection rate was around 90% confirmed by staining with anti GP pAb. The cells were stained with antibodies against HA-tag protein (green) and ERGIC or Golgi organelle markers (red), and the nuclei were stained with DAPI (blue). The mock-infected and transfected cells are shown as SFTSV ( −). **(f)** Co-localization analysis of HA-tagged N and mt-N proteins with Golgi apparatus markers shown in **(f)** using Fiji/ImageJ (***corresponds to a P value of < 0.001). Number of cells = n.

To confirm the importance of the interaction between vRNA and N protein in the localization of the N protein, a plasmid expressing a triple mutant N (mt-N) protein with substitution of three amino acids in the RNA-binding domain was constructed. Three amino acids (R64, K67, and K74) of the RNA-binding site were substituted with amino acid D, and pCAGGS-SFTSV mt-N was prepared^16^. The expression of mt-N protein was confirmed by western blotting (Fig. 3a). In a minigenome assay, the luciferase activity of mt-N protein was almost the same as that of the negative control, suggesting that the conformation of a functional RNP complex was inhibited by the mutation in the N protein (Supplementary Fig. 5).

An immunoprecipitation (IP) assay using an SFTSV anti-N antibody was conducted to investigate the interaction between N and minigenome RNA. First, the enhanced green fluorescent protein (eGFP) expression was confirmed by transfecting pATX-vRNA-eGFP into BSR-T7/5 cells (Fig. 3b). Following the IP assay, the presence of both N and mt-N proteins was confirmed by western blotting (Fig. 3c). Quantitative polymerase chain reaction PCR (qPCR) was performed by targeting the eGFP gene to identify if the N protein co-immunoprecipitated with minigenome RNA. The RNA copy number of the N protein was higher than that of the mt-N protein in the qPCR assay, which indicates the lack of mt-N binding to RNA (Fig. 3d).

The localization of mt-N protein in cells co-expressing RdRp, GP, and vRNA-Rluc was analyzed. The mt-N protein did not localize to the ERGIC and Golgi apparatus, in contrast to the functional N protein (Fig. 3e). HA-tagged N protein and mt-N protein were transfected into the SFTSV-infected BHK/T7-9 cells to understand the organization of N protein in infected cells. HA-tagged N protein was localized to the ERGIC and Golgi apparatus, whereas mt-N did not localize to these areas (Fig. 3f). The Mander’s correlation coefficient (MCC) M2 was used to evaluate the colocalization of green pixels to red pixels between the HA-N or HA-mt N protein to the ERGIC and Golgi apparatus (Fig. 3g). Its values are in the range from 0 to 1.0^14^. The HA-N protein showed a significant correlation with the ERGIC, and Golgi apparatus compared with the mt-N protein. Interestingly, the incidence of N protein localization to the ERGIC was higher than that to the Golgi apparatus in both N and mt-N proteins which agrees with the results of transfection experiments. These results show that the interaction between the N protein and viral RNA is an indispensable factor for viral replication.

## Discussion

Negative-strand RNA viruses require a host cell secretory pathway for viral infection; the transportation of cellular proteins and cargo from the Golgi apparatus to the ER via the ERGIC has been reported^9,17^. Viruses belonging to the order *Bunyavirales* use not only the ER and Golgi apparatus, but also the ERGIC as site of virion assembly^12,18,19^. The localization of viral structural proteins is indispensable for infectious viral particle formation^9,15,18,20–22^. Previous studies showed that RdRp was partially localized to the ERGIC and Golgi apparatus in the presence of GP, whereas N protein was widely expressed in the cytoplasm, even in cells coexpressing GP^13^. In this study, we showed that the interaction between the N protein and viral RNA plays a critical role in the intracellular transportation of N protein to the ERGIC and Golgi apparatus. Thus, N protein translated in the cytoplasm forms an RNP complex together with vRNA to further interact with GP localized in the ERGIC via RdRp to be taken up by virus particles.

In Bunyavirus particle formation, the N protein is essential for the transcription and replication of vRNA along with RdRp^23^.Multiple copies of the N protein coat each segment of vRNA forming, together with the L protein by with one RdRp, a functional RNP complex. The N protein coating of the vRNA is thought to play a role in protecting vRNA from the host defense mechanisms. The RNP complex interacts with GP in RNP packaging to be incorporated into the envelope and forms particles in the Golgi apparatus in which GP is accumulated^24–26^. The GP of the Uukuniemi virus is translated by ribosomes on the ER and that viral particle formation occurs in the ERGIC and budding continues in the Golgi apparatus^12^. Moreover, ERGIC is an important organelle for intracellular transport and particle formation of bunyaviruses and that N proteins are transported by the action of microtubules^12,15^. Lundu et al. reported that viral structural proteins in SFTSV-infected cells were localized in the ERGIC and Golgi apparatus, which are important cell components for virion formation^13^. Furthermore, the Gn of Rift Valley fever virus is localized in the Golgi apparatus alone, whereas RdRp is localized together with Gn in the Golgi apparatus^21^. Similarly, in SFTSV, only GP was localized in the ER, ERGIC, and Golgi apparatus, but RdRp was partially localized in the ERGIC and Golgi apparatus in the presence of GP. By contrast, N protein was not localized in the ERGIC and Golgi apparatus, even in the presence of GP^13^. These findings suggest that N protein is not directly bound to GP or RdRp, but is necessary for translocation to the ERGIC or Golgi apparatus for virion formation.

In the present study, the viral genome was simultaneously expressed with viral structural proteins. vRNA-Rluc or vRNA-eGFP was used as an alternative to vRNA to conduct experiments safely at the BSL2 facility. As a result, it was confirmed that the N protein, RdRp, GP, and RNA mimicking vRNA, when simultaneously introduced into the cells, resulted in the localization of the N protein in the ERGIC and Golgi apparatus. These results suggest that localization of the N protein requires association with RdRp and vRNA. The results of our study are consistent with a previous study showing that N protein forms a multimer in the RNP complex and wrapping around the vRNA, and with the RdRp interactions with the vRNA which occurs by direct contacts with the 5′ and 3′ ends of the vRNA^27,28^. On the contrary, in any other combination of viral structural proteins with the viral genome, the N protein could not be fully localized to the ERGIC nor to the Golgi apparatus.

In the confocal microscopy images, localization of the N protein in the presence of vRNA-Rluc was not detected in the ERGIC and Golgi apparatus. In contrast, luciferase minigenome activity was detected, and the level of activity was higher than the level of luciferase activity with GP (Fig. 2c). This finding indicated that even though the RNP complex functions as a transcription complex in the cell cytoplasm when it interacts with GP in RNP packaging in the ERGIC or Golgi apparatus, this mechanism might be affected by other cellular factors, which requires further investigation.

Mutations involved in the binding of SFTSV N protein to vRNA have been identified (R64/D, K67/D, K74/D)^16^. In this study, we used an mt-N protein with the substitution of three amino acids that are important for the binding of N protein to vRNA. In addition, Jiao et al. did not verify the lack of mt-N binding to RNA. Therefore, an IP assay using an anti-N antibody was conducted to investigate the binding between the N protein and the minigenome RNA. As a result, we were able to provide evidence of the mt-N protein’s lack of ability to bind to RNA. Due to the same reason, the mt-N protein could not be sorted to the ERGIC and Golgi apparatus in the presence of RdRp, GP, and vRNA. This finding suggests that the interaction between the N protein of SFTSV and vRNA is important for the localization of viral proteins. On the contrary, unexpected structural defects in the N protein caused by mutations other than the lack of RNA-binding ability could inhibit RNP assembly. These results indicate that proper interaction of the N protein with vRNA and RdRp is required for the transcription of the RNP complex and for localization of structural proteins to the ERGIC and Golgi apparatus.

To distinguish between the orderly process of natural infection and overexpression phenomenon in transfection experiments, we conducted experiments using HA-tagged N and mt-N proteins. This led us to understand the sorting of N proteins in infected cells. HA-tagged N protein was localized to the ERGIC and Golgi apparatus, whereas the mt-N protein did not localize to these areas. In the overexpressed systems shown in this study, abundant accumulation was observed in the ERGIC but not in the Golgi apparatus. Also, the similar pattern was observed in the natural infection. Unfortunately, we were unable to perform a meticulous study to determine the distribution of N proteins in all cellular compartments. Further investigations are needed to understand the differences and/or similarities between the overexpression system and process of natural infection.

In some enveloped viruses, such as murine leukemia virus and Ebola virus, an interaction between the viral RNP complex and envelope proteins facilitates the budding of virus particles^29,30^. In some other enveloped viruses such as coronavirus and Marburg virus, RNPs play a critical role in the viral envelope formation and virus particle production ^31,32^.

In the IP assay, we attempted to demonstrate the binding of RdRp to form the RNP complex; however, we were unable to obtain a western blot image that could withstand publication. Thus, the results of this study do not convincingly show the formation of an RNP complex or the exact interaction between GP and the RNP complex. However, the cytoplasmic tails of GP of Uukuniemi virus can directly interact with N protein to enable the packaging of RNPs into virus particles^33^. Therefore, further studies are needed to understand the interaction between Gn/Gc and the N proteins of SFTSV.

Overall, the findings of this study will shed light on the basic process of the SFTSV life cycle. This will be useful for developing specific inhibitors interfering with N protein and vRNA and antiviral strategies against SFTSV.

In conclusion, we focused solely on the nuclear protein N protein and analyzed the interaction between vRNA, and other viral proteins required for intracellular localization of N protein. Further studies are needed to clarify the details of intracellular pathways such as N protein or RdRp synthesis and RNP complex formation that occurs in the cytoplasm. Such studies will lead to a full understanding of the SFTSV life cycle and virus particle formation.

## Materials and methods

### Cells

Vero E6 cells (ATCC C1008) were maintained in Eagle’s minimum essential medium (EMEM) (Gibco; Thermo Fisher Scientific, MA, USA) supplemented with 5% heat-inactivated fetal bovine serum (FBS) (Biowest, Nuaillé, France), 1% MEM non-essential amino acids (Gibco), 1% insulin-transferrin-selenium (Gibco; Thermo Fisher Scientific), 1% penicillin (50 units/mL), streptomycin (50 μg/mL; Sigma-Aldrich Co., St Louis, MO, USA), and 1% gentamicin (100 μg/mL; Sigma-Aldrich). 293 T cells were maintained in Dulbecco’s Modified Eagle’s Medium (DMEM; Sigma-Aldrich) supplemented with 10% FBS, 1% penicillin, and streptomycin. BHK/T7-9 (RRID: CVCL_A8V7) cells stably expressing the T7 RNA polymerase gene under the control of the beta-actin promoter were kindly provided by Dr. Naoto Ito and Dr. Makoto Sugiyama from Gifu University^34^. The cells were maintained in EMEM supplemented with 5% FBS, 1% penicillin, streptomycin, and 10% tryptone phosphate broth (TPB; Thermo Fisher Scientific). BSR-T7/5 cells stably expressing T7 RNA polymerase were kindly provided by Dr. K. K. Conzelmann^35^ (Max-von-Pettenkofer Institut, Munich, Germany). The cells were maintained in DMEM supplemented with 10% FBS, 10% TPB, and antibiotics (1 mg/mL Geneticin (G418) (Nacalai Tesque, Kyoto, Japan) or 1% penicillin and streptomycin). All cells were cultured in a 5% CO_2_ incubator at 37 °C.

### Virus

The SFTSV YG1 strain was used in this study^2^. Experiments involving viral infections were performed in a biosafety level 3 (BSL-3) facility at the Institute for Genetic Medicine, Hokkaido University. The Vero E6 cells were seeded at 5 × 10^3^ cells/well on 12-well glass slides (Matsunami Glass Ind., Ltd. Osaka, Japan) and incubated at 37 °C in a 5% CO_2_ incubator for 16 h. The cells were inoculated with virus at a multiplicity of infection of 0.1. Twenty hours after inoculation, the slides were fixed with 4% paraformaldehyde phosphate buffer solution (PPBS) (FUJIFILM Wako Pure Chemical Co., Osaka, Japan) for 20 min at room temperature.

### Plasmids

The full open reading frames of SFTSV N protein, GP, and RdRp were previously cloned into the pCAGGS-MCS mammalian expression vector as described by Lundu et al. and were named pCAGGS-SFTSV N, pCAGGS-SFTSV GP, and pCAGGS-SFTSV L, respectively^13^. A triple mutant plasmid was prepared to introduce three amino acid mutations (R64/D, K67/D, and K74/D)^16^ into the conserved region of the N protein sequence (GenScript, Tokyo, Japan). The synthesized gene was cloned into the pCAGGS-SFTSV N plasmid and named pCAGGS-SFTSV mt-N. As an alternative to the viral genome, a minus-sense reporter gene, Renilla luciferase (vRNA-Rluc) or eGFP, which mimics the viral genome with untranslated regions of the M segment at both ends using pATX expression vectors named pATX-vRNA-Rluc and pATX-vRNA-eGFP were prepared^36^. Epitope-tagged SFTSV N and mt-N were constructed using primers containing the hemagglutinin (HA) tag sequence as explained elsewhere^13^. The plasmid sequences were confirmed by performing a Sanger sequencing analysis before use.

### Expression of recombinant proteins and minigenome

To analyze the subcellular localization of SFTSV structural proteins, the N protein, mt-N protein, GP, RdRp, vRNA-Rluc and T7 polymerase were expressed in various combinations in Vero E6 cells. Briefly, the Vero E6 cells (5 × 10^3^ cells/well) were seeded on 24-well slides (Matsunami) and incubated at 37 °C in a 5% CO_2_ incubator for 16 h. The cells were transfected using a TransIT-LT1 reagent (Mirus Bio LLC, Madison, WI, USA) with a single expression plasmid or combinations of expression plasmids, including pCAGGS-SFTSV N, pCAGGS-SFTSV mt-N, pCAGGS-SFTSV GP, pCAGGS-SFTSV L, pATX-vRNA-Rluc, pATX-vRNA-eGFP, and pCAGGS-T7, according to the manufacturer’s protocol. After 24 h of incubation, the cells were fixed with 4% PPBS (FUJIFILM).

### Minigenome assay

BHK/T7-9 cells (1 × 10^5^ cells/well) were seeded in a 12-well plate and incubated for 16 h in a 5% CO_2_ incubator. The cells were transfected with either pCAGGS-SFTSV N or pCAGGS-SFTSV mt-N together with pCAGGS-SFTSV L, pATX-vRNA-Rluc, and a firefly luciferase expression plasmid for the standardized measurement of gene transfer^37^. The pCAGGS/MCS plasmid without any insertion was used as a negative control. All transfected cells were incubated at 37 °C in a 5% CO_2_ incubator for 24 h. The culture supernatant of each well was removed, and the cell pellet was lysed using 20 µL of passive lysis buffer (Promega Co., Madison, WI, USA). The cell lysate was reacted with Dual-Luciferase Reporter assay reagents (Promega Co.), and the Renilla luciferase and firefly luciferase activities were measured using the GloMax-Multi Detection System (Promega Co.). Polymerase activity was determined based on the ratio of Renilla luciferase activity to firefly luciferase activity.

All data were presented as mean ± standard deviation. Statistical analysis was performed using a one-tailed Student’s t-test. A P-value of > 0.01 was considered significant.

### Indirect immunofluorescence assay

Formaldehyde-fixed cells were permeabilized with 1% Triton X-100 in PBS for 10 min and blocked with 1% bovine serum albumin (BSA)/PBS for 30 min. The cells were labeled with rabbit anti-N synthetic peptide (40–54) polyclonal antibody (pAb) (Sigma-Aldrich) or YG1.7-3-3-4 mouse anti-N monoclonal antibody (mAb) (kindly provided by Prof. Ayato Takada, Hokkaido University), rabbit anti-Gn (#6647) and anti-Gc (#6653) pAb (Prosci Inc., Poway, CA, USA), and rabbit anti-L synthetic peptide (386–400) pAb (Frontier Laboratories, Fukushima, Japan). The organelles were marked with mouse anti-ERGIC-53 (G1/93) mAb IgG1 (Enzo Life Sciences Inc., NY, USA) or rabbit anti- ERGIC53 pAb (Proteintech, IL, USA) and anti-GM130 ab-cis Golgi marker ab 169276 (Abcam, Cambridge, UK). Alexa Fluor 488 anti-rabbit IgG, Alexa Fluor 594 anti-rabbit IgG, and Alexa Fluor 594 anti-mouse IgG (Invitrogen; Thermo Fisher Scientific) were used as secondary antibodies. Anti-HA-tag pAb labeled with Alexa Fluor 488 (Medical & Biological Laboratories Co., Ltd, Nagoya, Japan) was used to label the HA-tag. The stained cells were mounted with ProLong Gold Antifade reagent with 4′6-diamidino-2-phenylindole (DAPI) (Invitrogen; Thermo Fisher Scientific) according to the manufacturer’s instructions. Fluorescent images were acquired using an FV1000-D Olympus confocal microscope (Olympus Co., Tokyo, Japan) with a 60 × oil lens.

### Image analysis

Image and statistical analyses of colocalization were performed using the open-source Fiji/ImageJ software (https://imagej.net). PCC and MCC M2 were obtained using the ImageJ plugin “Coloc2.”

### Immunoprecipitation(IP) assay

BSR-T7/5 cells were transfected with pCAGGS-SFTSV N or pCAGGS-SFTSV mt-N together with pCAGGS-SFTSV L and pATX-vRNA-eGFP, according to the manufacturer’s instructions, and incubated at 37 °C in a 5% CO_2_ incubator for 24 h. The cells (5 × 10^5^) were rinsed once with ice-cold PBS followed by lysis in 0.5 mL of polysome lysis buffer containing 20 mM Tris–HCl (pH 7.5), 100 mM KCl, 5 mM MgCl_2_, 0.5% Nonidet P-40, 100 U/mL RNase inhibitor, and 1 × protease inhibitor cocktail. The polysomal lysate was incubated for 20 min on ice to obtain the supernatant after centrifugation. The SFTSV N or SFTSV mt-N protein was immunoprecipitated using Dynabeads Protein A Immunoprecipitation Kit (Thermo Fisher Scientific) according to the manufacturer’s protocol. Briefly, the binding antibody used was 5 µg of mouse anti-N mAb and mouse IgG2b, kappa monoclonal [MPC 11]—isotype control (Abcam)—incubated with magnetic beads with rotation for 10 min at room temperature. Subsequently, 250 µL of the polysomal lysate was added to the magnetic bead-Ab complex, followed by several washing steps, and incubated with rotation for 1 h at 4 °C. The supernatant was removed from the magnetic beads-Ab-Ag complex after washing, and 1 mL of Isogen (Nippon Gene, Tokyo, Japan) or 20 µL of 2 × SDS sample buffer was added directly into the complex, depending on the following experiment.

### Quantitative PCR assay

Total RNA was extracted from the samples acquired from the IP assay according to the manufacturer’s protocol and used as a template for reverse-transcription (RT-PCR). RT-PCR was performed using ReverTra Ace qPCR RT Master Mix (Toyobo, Osaka, Japan). The KAPA SYBR FAST for LightCycler 480 Kit (Sigma Aldrich) was used to perform the qPCR reaction using the following primer set: forward primer: 5′-CAAGGAGGACGGCAACATCCTGGG-3′ and reverse primer: 5′-ATGCCGTTCTTCTGCTTGTCGGCC-′3-expressing eGFP gene. qPCR was performed using LightCycler 480 II (Roche Applied Science, Oberbayern, Germany).

### Western blotting

HEK293T cells were seeded on a six-well plate at 3 × 10^5^ cells/well and incubated overnight at 37 °C in a 5% CO_2_ incubator. The cells were transfected with pCAGGS-SFTSV N, pCAGGS-SFTSV mt-N, or pCAGGS/MCS vector as a negative control and cultured for 24 h. The cells were rinsed once with PBS and lysed by treatment with 4 × Laemmli buffer (Bio-Rad Laboratories, CA, USA) supplemented with 2-mercaptoethanol. The collected cell lysate and samples from the IP assay were incubated at 100 °C for 5 min. The prepared samples were subjected to sodium dodecyl sulfate–polyacrylamide gel electrophoresis (SDS-PAGE) using 5–20% gradient gels (ATTO Co., Tokyo, Japan). After electrophoresis, the proteins were transferred to a 0.45-µm pore immunoblot PVDF membrane (Millipore, Billerica, MA, USA). After blocking the membrane with Block Ace (Dainippon Pharma Co., Osaka, Japan) supplemented with 0.1% BSA for 1–16 h at 4 °C, antibody treatment was performed. A rabbit anti-N pAb diluted 1:1000 in Can Get Signal Immunoreaction Enhancer Solution 1 (Toyobo) was used as the primary antibody and was incubated for 1 h at room temperature. Horseradish peroxidase-conjugated protein A (Prozyme Inc., CA, USA) at a 3000-fold dilution or horseradish peroxidase-conjugated mouse anti-rabbit IgG as the secondary antibody (Jackson ImmunoResearch Laboratories Inc., Baltimore, MD, USA), diluted 10,000 times with Can Get Signal Immunoreaction Enhancer Solution 2 (Toyobo) was used as the secondary antibody and incubated for 1 h at room temperature. After washing with PBS, the proteins were reacted with Amersham ECL Prime (GE Healthcare Life Science, PA, USA) and detected using ImageQuant LAS 4000 mini (GE Healthcare Life Science) according to the manufacturer’s protocol.

## Supplementary Information

Supplementary Figures.

## Data Availability

The datasets generated during the current study are available from the corresponding author upon reasonable request.
