# Supplementary material for: Subcellular localization of nucleocapsid protein of SFTSV and its assembly into the ribonucleoprotein complex with L protein and viral RNA
Source: Sci Rep. 2021 Nov 26;11:22977. doi: 10.1038/s41598-021-01985-x (PMC8626419; doi:10.1038/s41598-021-01985-x)
Supplement: Supplementary file 1 — Supplementary Figures. [file 41598_2021_1985_MOESM1_ESM.pdf]

Subcellular localization of nucleocapsid protein of SFTSV and its assembly into the ribonucleoprotein complex with L protein and viral RNA

Sithumini M.W. Lokupathirage,[1] Yoshimi Tsuda,[2,3] Kodai Ikekame,[3] Kisho Noda,[4] Devinda S. Muthusinghe,[1] Fumiya Kozawa,[4] Rashid Manzoor,[5] Kenta Shimizu,[2,3] and Kumiko Yoshimatsu[1,6*]

[1]Graduate School of Infectious Diseases, Hokkaido University, Sapporo 060-8638, Japan
[2]Department of Microbiology and Immunology, Faculty of Medicine, Hokkaido University, Sapporo 060-8638, Japan
[3]Graduate School of Medicine, Hokkaido University, Sapporo 060-8638, Japan
[4]School of Medicine, Hokkaido University, Sapporo 060-8638, Japan
[5]International Institute for Zoonosis Control, Hokkaido University, Sapporo 001-0020, Japan
[6]Institute for Genetic Medicine, Hokkaido University, Sapporo 060-0815, Japan

*Corresponding author:
yosimatu@igm.hokudai.ac.jp

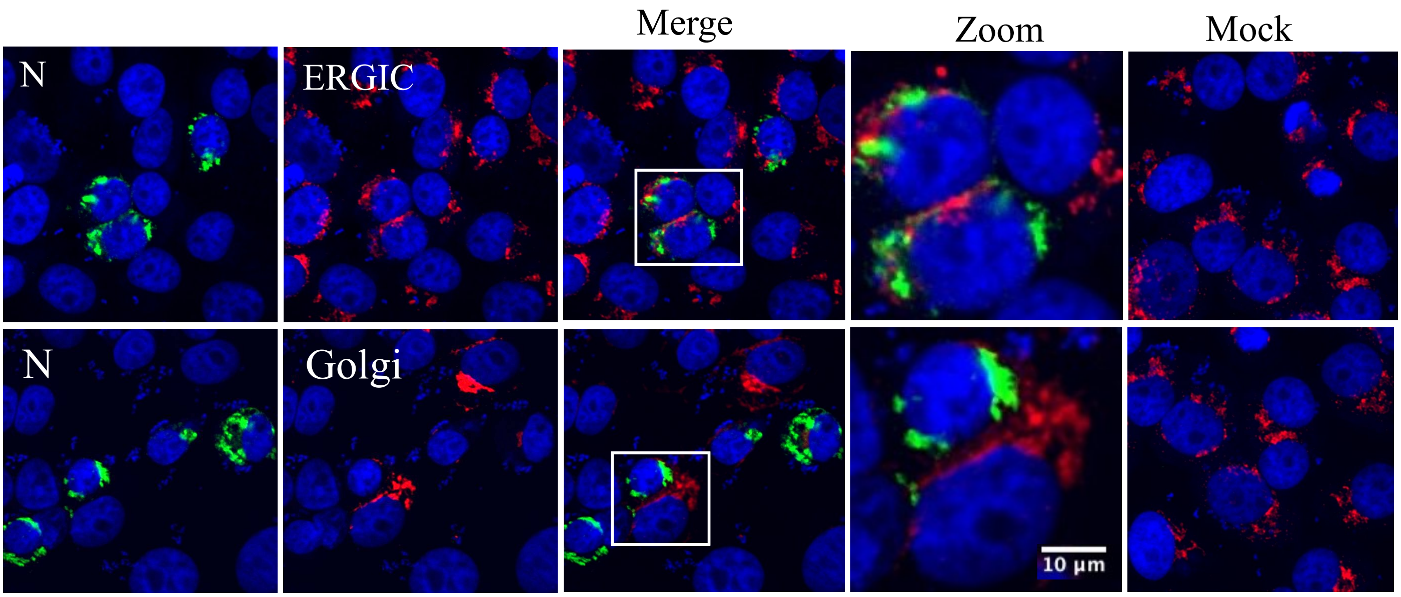

Supplementary figure. 1: Subcellular localization of N protein in GP transfected cells.

Effect of GP on localization of N protein in Vero E6 cells. Vero E6 cells were transfected with pCAGGS-SFTSV-N and pCAGGS-SFTSV-GP and fixed at 24 hours post-transfection. The cells were stained with antibodies against N protein (Green) and ERGIC or Golgi organelle markers (Red), and nuclei were stained with DAPI (Blue). Magnification of the merged area is shown on the right side of the merged image. Mock-transfected cells are shown as mock images.

(a)

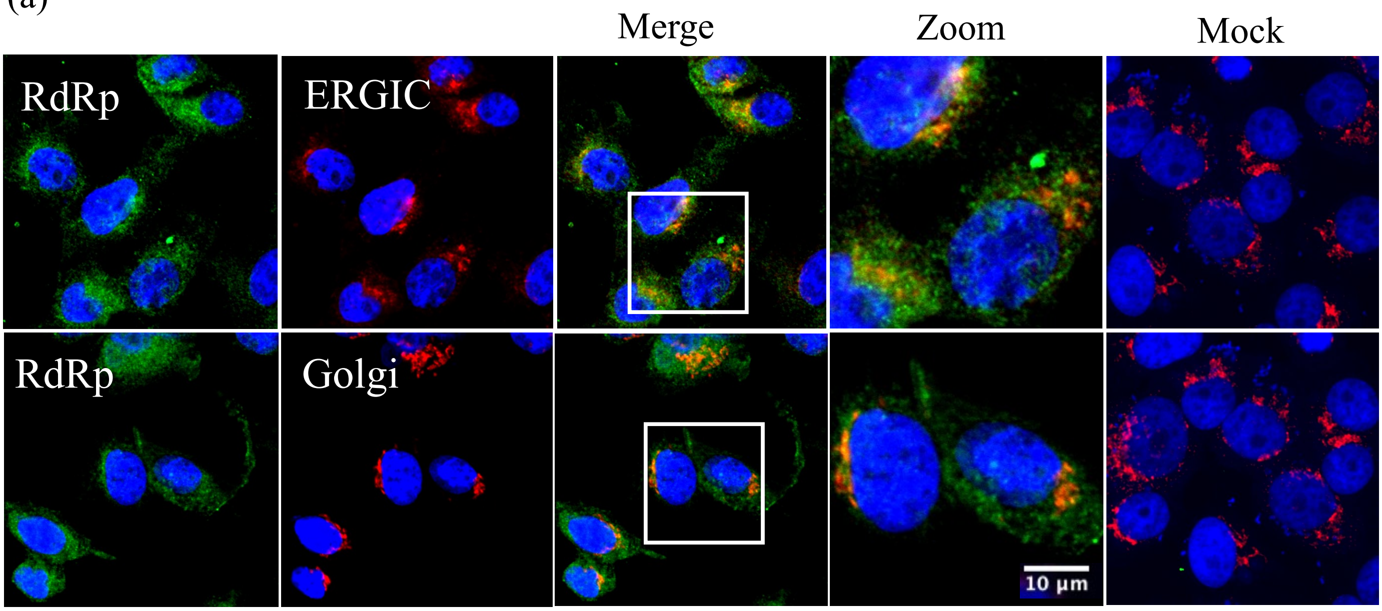

(b)

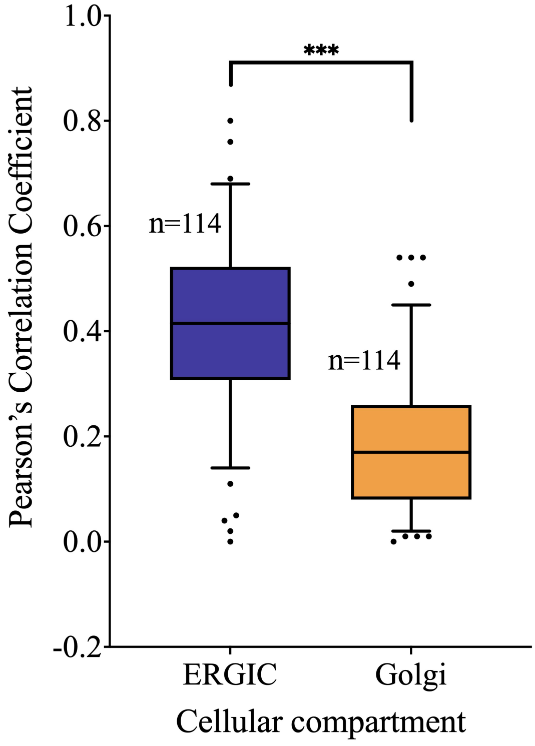

Supplementary figure 2: Subcellular localization of RdRp proteins in transfected cells.

(a) Cellular localization of RdRp in GP and RdRp co-expressed Vero E6 cells. The cells were stained with antibodies against RdRp (Green) and ERGIC or Golgi organelle markers (Red), and nuclei were stained with DAPI (Blue). The yellow areas in the merged image show the colocalization of proteins with the organelle markers. Magnification of the merged area is shown on the right side of the merged image. Mock-transfected cells are shown as mock images.

(b) Co-localization analysis of RdRp and ERGIC or Golgi apparatus by Fiji/ImageJ. Number of Samples= n

Supplementary Figure 2, Lokupathirage *et al.*

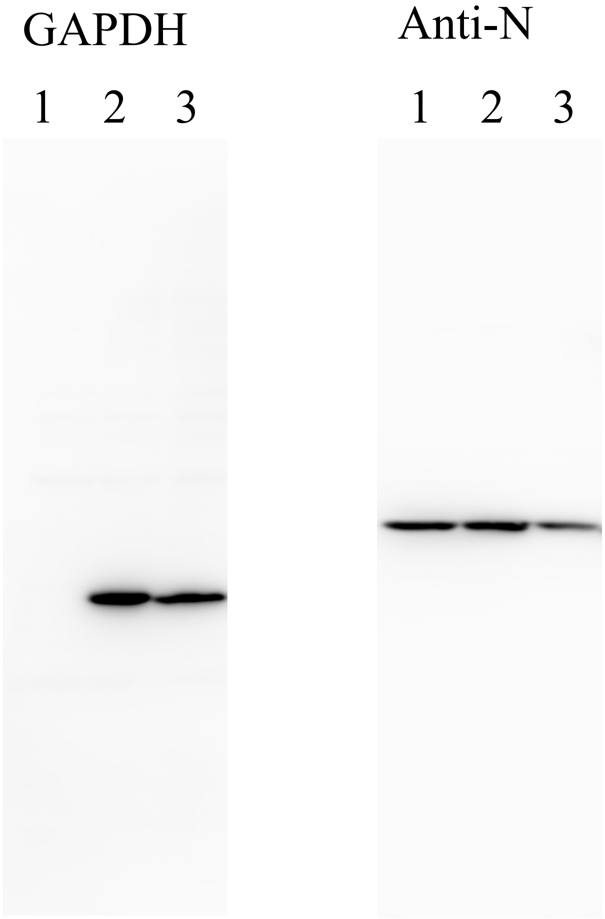

	Lane 1	Lane 2	Lane 3
Transfection	mock	wt-N	mt-N

Supplementary figure 3: Full-size image of Fig. 3a

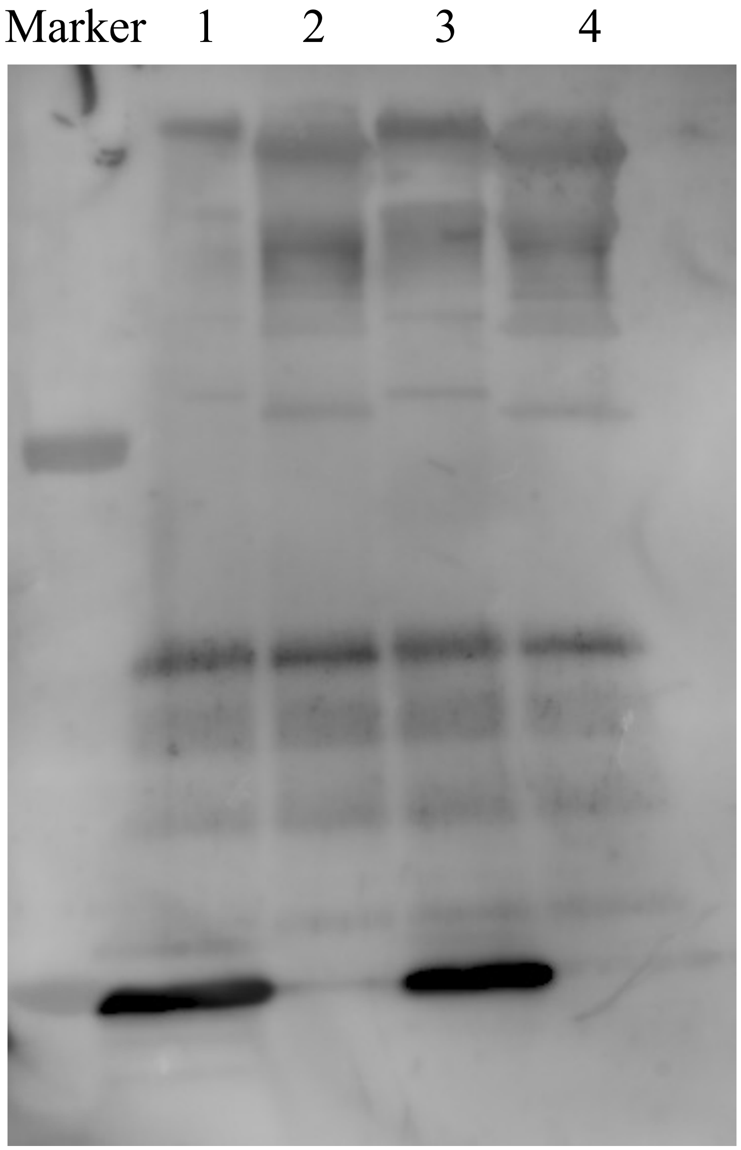

	Lane 1	Lane 2	Lane 3	Lane 4
Transfection	wt-N		mt-N	
Antibody	Anti-N	Isotype control	Anti-N	Isotype control

Supplementary figure 4: Full-size image of Fig. 3c

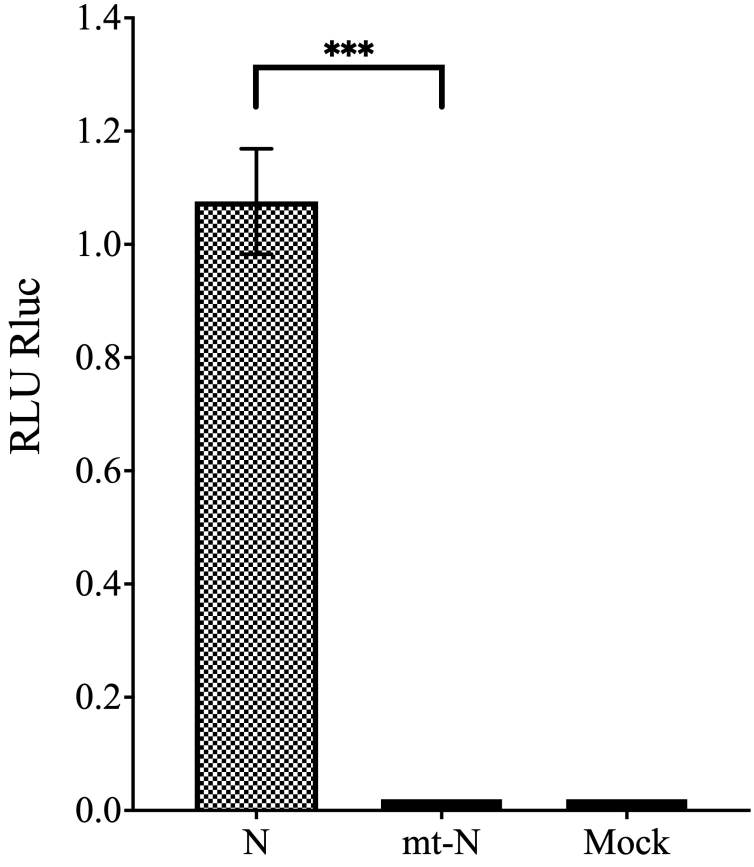

Supplementary figure 5. Expression and functionality of mt-N protein of SFTSV.
Detection of transcriptional activity by a minigenome assay. N protein or mt-N protein was transfected into BHK/T7-9 cells together with RdRp and vRNA-Rluc, and the luciferase activities were compared. Mock-transfected cells are shown as mock images. (**corresponds to a P-value < 0.01.)
